# WORKING LIFE EXPECTANCY AMONG MIDDLE-AGED AND OLDER MEXICAN MEN AND WOMEN

**DOI:** 10.1093/geroni/igae098.1418

**Published:** 2024-12-31

**Authors:** Chi-Tsun Chiu

**Affiliations:** Academia Sinica, Taipei, Taipei, Taiwan (Republic of China)

## Abstract

With rapid population aging, partly contributed by increasing life expectancy, work and retirement related issues – including increases in retirement age and sustainability of pension systems – are increasingly pertinent. Many countries have increased or considered increasing the legal age of retirement for receipt of pensions and introduced measures for increasing the length of working life – in Mexico specifically, from 2028 onward, early retirement will be penalized. Furthermore, more individuals are continuing to work beyond current mandatory retirement ages, such as being self-employed or working in the gig economy. Estimates of the average number of years individuals remain in the workforce from middle age (age 50 years) are needed to assess the feasibility of potential policy changes and provide the evidence base to inform policymaking. This study uses the Mexican Health and Aging Study (MHAS), a national longitudinal study of adults 50 years and older in Mexico, to compute life expectancy (LE) and working life expectancy (WLE) by gender. Our results show that at age 50, Mexican men’s LE is 29.0 (27.8-30.2) and WLE is 16.7 (16.0-17.4) years, i.e. 57.6% of LE. Mexican women live longer, with a LE of 32.4 (31.5-33.3) years, however, have a much lower WLE of 8.1 (7.5-8.8) years or 25.0% of LE. The findings highlight the strong sex disparity in WLE, disfavoring women, in Mexico and underscore the need for policymakers to address it. Targeted policies and initiatives are required to promote greater and continued labor force participation for middle-aged and older Mexican women.

